# Development and Validation of the Kazakhstan Version of the Questionnaire Based on the Telehealth Usability Questionnaire and Model for Assessment of Telemedicine Models for Evaluating the Usability and Effectiveness of Telemedicine Services Among Physicians: Multiphase Cross-Sectional Study

**DOI:** 10.2196/80693

**Published:** 2026-02-02

**Authors:** Kulzhamila Kenessova, Saule Tuktibayeva, Myrzabek Rysbekov, Abay Baigenzhin, Aigul Sultangaziyeva, Bakhytzhan Alimov

**Affiliations:** 1 Department of Public Health and Healthcare, South Kazakhstan Medical Academy, Al-Farabi Square, 1, Shymkent, Kazakhstan, 7 7058528373; 2 Department of Pediatrics, Akhmet Yassawi University, Medical Center Shubarsu Clinic, Shymkent, Kazakhstan; 3 Tsoy Scientific and Educational Center of Surgery, Astana Medical University, Astana, Kazakhstan; 4 JSC National Scientific Medical Center, Astana, Kazakhstan; 5 Department of Academic Quality, Asfendiyarov Kazakh National Medical University, Almaty, Kazakhstan

**Keywords:** telemedicine, digital health, questionnaire validation, Telehealth Usability Questionnaire, TUQ, Model for Assessment of Telemedicine, MAST, Kazakhstan, physicians

## Abstract

**Background:**

Kazakhstan has lacked validated tools to comprehensively assess physicians’ perceptions, usability, and perceived effectiveness of telemedicine services. International frameworks such as the Telehealth Usability Questionnaire (TUQ) and the Model for Assessment of Telemedicine (MAST) have not previously been adapted to the national clinical and organizational context.

**Objective:**

This study aims to develop and validate TUQ-MAST-KZ, a Kazakhstan-adapted questionnaire integrating components of the TUQ and MAST models to assess physicians’ perceptions, usability, and effectiveness of telemedicine services.

**Methods:**

A multiphase study was conducted, including literature review, questionnaire development, linguistic and cultural adaptation, expert content validity assessment, and pilot testing. An online survey (Google Forms) was administered to 156 physicians representing different regions and levels of health care delivery in Kazakhstan. Internal consistency (Cronbach α) and content validity indices were calculated. Additional evaluations covered clarity, structure, and practical applicability.

**Results:**

The final TUQ-MAST-KZ instrument contains 27 items capturing technological, clinical, organizational, and behavioral dimensions of telemedicine use. The scale demonstrated high content validity (scale-level content validity index=0.94). Internal consistency was excellent, with an overall Cronbach α of 0.924. Respondents reported that the questionnaire was clearly structured, easy to complete, and relevant to clinical practice. Organizational items identified key barriers to telemedicine adoption, including limited infrastructure, insufficient managerial support, and the need for additional training.

**Conclusions:**

TUQ-MAST-KZ is a valid, reliable, and practice-oriented instrument for assessing physicians’ perceptions of telemedicine services in Kazakhstan. It can support digital health monitoring, implementation analysis, educational planning, and policy development. Future studies should evaluate its applicability across broader samples and diverse clinical specialties.

## Introduction

Telemedicine, which originated in the 1950s, has evolved into one of the most rapidly developing components of modern health care due to advances in technology and support from governmental and international organizations [1]. Its importance increased markedly during the COVID-19 pandemic, when remote technologies ensured continuity of medical care despite limited access to in-person consultations [2]. The effectiveness of telemedicine has been confirmed by systematic reviews demonstrating its positive impact on health care accessibility, clinical outcomes, and patient satisfaction [3].

According to the World Health Organization (WHO), telemedicine refers to providing health care services at a distance using information and communication technologies to transmit reliable information needed for diagnosis, prevention, treatment, and the education of health care professionals [4]. The WHO reports that the development of telemedicine in Kazakhstan began in the early 2000s. The country now operates a National Telemedicine Network that connects regional and district-level medical organizations. This system has significantly improved access to specialized medical care in rural and remote areas and has strengthened the capacity of health care personnel through remote consultations and training [5].

In line with Kazakhstan’s national digital health policy, the development of telemedicine is viewed as one of the key priorities in the modernization of the health care system. According to the Healthcare Development Concept of the Republic of Kazakhstan until 2026, “telemedicine service delivery systems will be aligned with international standards, including an expanded range of diagnostic services and the use of modern digital solutions” [6]. Achieving this alignment requires not only technological modernization but also the introduction of systematic tools for assessing the quality and perception of telemedicine services. For the successful implementation of telemedicine, it is essential to systematically identify its limitations and strategies for overcoming them. This requires the use of structured instruments that enable a comprehensive assessment of the effectiveness, accessibility, safety, economic viability, and perception of telemedicine services from the perspectives of both patients and health care professionals. As noted by Hajesmaeel-Gohari and Bahaadinbeigy [7], the availability of such tools provides an evidence-based foundation for analyzing the strengths and weaknesses of telemedicine programs and supports the development of informed policies for their implementation.

A similar need for standardization was highlighted by Doraiswamy et al [8], who, in their scoping review of 543 publications, emphasized the importance of unified tools and terminology for evaluating the impact of telemedicine, particularly in resource-limited settings. The absence of standardized approaches restricts the ability to conduct cross-country comparisons and hinders the development of a robust evidence base for evaluating telemedicine effectiveness.

According to Gullslett et al [9], telemedicine has reached a stage of maturity in most European countries. Nevertheless, substantial barriers persist, including insufficient funding, infrastructural limitations, and the lack of systematic monitoring. Only 37% of countries conduct regular assessments of telemedicine service quality, reflecting global challenges in digital maturity and limiting the potential for cross-national data comparison [9]. These findings underscore the need to develop national tools for systematic telemedicine assessment, particularly in countries undergoing active digital health expansion, such as in Kazakhstan.

Despite the progress achieved, systematic research evaluating the perception, effectiveness, and barriers to the implementation of telemedicine remains limited. In this context, the development of nationally adapted instruments capable of providing a comprehensive assessment of telemedicine services and their acceptance within the medical community is particularly important. A systematic review by Kruse et al [10] identified key barriers to the adoption of telemedicine, including resistance to change, insufficient staff training, and the lack of standardized evaluation frameworks. These findings highlight the need for comprehensive and validated assessment tools.

This study analyzes the most commonly used questionnaires for evaluating telemedicine and remote health care technologies. Their selection was based on their frequency of use in systematic reviews and on their relevance to key dimensions of telemedicine evaluation, including perception, usability, and effectiveness.

In the context of large-scale digital health implementation, it is particularly important to use validated instruments capable of capturing the subjective experiences of users and the perspectives of health care professionals. Quantitative indicators—such as the number of consultations or measures of cost-effectiveness—do not fully capture how telemedicine is integrated into clinical practice or how it influences clinical decision-making, both of which are critical for systems with varying levels of digital maturity. Over the past decades, numerous instruments have been developed to assess satisfaction with and perceptions of telemedicine services, including the Client Satisfaction Questionnaire and the Patient Satisfaction Questionnaire, which assess overall patient satisfaction with medical care; the Questionnaire for User Interaction Satisfaction, the Post-Study System Usability Questionnaire, and the System Usability Scale, which evaluate usability and user interaction with digital systems; instruments based on the technology acceptance model, which focus on perceived usefulness and technology acceptance; as well as telemedicine-specific questionnaires such as the Telemedicine Perception Questionnaire, the Telemedicine Satisfaction Questionnaire (TSQ), the Telemedicine Satisfaction and Usefulness Questionnaire, the Patient Assessment of Communication during Telemedicine Questionnaire, and the Service User Technology Acceptability Questionnaire, all of which assess specific aspects of perception, satisfaction, and communication in telemedicine services [11-21]. However, most existing instruments are patient-oriented and assess only selected aspects of interaction—such as usability, communication, satisfaction, perceived usefulness, and technology acceptance—while the professional and organizational context, which is essential for physicians, is addressed to a limited extent. As a result, these scales lack a comprehensive assessment that encompasses both user-focused dimensions and the specific characteristics of clinical practice and conditions of telemedicine implementation, which is consistent with the findings of previously published review studies [7].

In this context, instruments specifically designed to assess health care professionals’ perceptions of telemedicine remain scarce, even though physicians’ perspectives play a crucial role in the successful implementation and overall effectiveness of telemedicine solutions. This gap underscores the need for validated assessment tools tailored to the professional community. Despite differences among existing instruments, their limitations are systemic and manifest in similar ways: a predominant focus on patients, the assessment of isolated components of interaction, and the absence of measures capturing professional and organizational aspects that are essential for physicians.

In recent scientific literature, several new instruments have been developed to assess the perception and organization of telemedicine services; however, their use remains limited. One such tool is the questionnaire by Cho et al [22], designed to characterize telemedicine programs in primary care. Despite its relevance and structured approach, it is not based on a theoretical model, has not undergone psychometric validation, and is used primarily for research purposes, which restricts its systematic application. Similar limitations are observed in the 2024 instruments: the Chinese tool developed by Du and Gu [23], which focuses exclusively on patients, and the German questionnaire by Traulsen et al [24], which was applied only in nursing homes. Both tools are highly specialized, lack multilevel validation, and are not intended for assessing telemedicine perceptions among health care professionals.

Thus, most contemporary instruments developed after 2020 have limited or one-time applicability, underscoring the need for a universal, validated, and practice-oriented tool for assessing physicians’ perceptions, usability, and the effectiveness of telemedicine services. The Telehealth Usability Questionnaire (TUQ) questionnaire, developed by Parmanto et al [25] in 2016, is one of the most versatile and methodologically grounded instruments for evaluating telemedicine systems. Its structure incorporates elements from the technology acceptance model, TSQ, and Post-Study System Usability Questionnaire models, enabling a comprehensive assessment of user-related aspects such as usability, effectiveness, and satisfaction. Its high internal consistency (Cronbach *α*>0.93) and successful use in both video consultations and mobile apps confirm its reliability and broad applicability [7]. However, despite its psychometric robustness and broad coverage of user characteristics, the TUQ primarily reflects user and technological dimensions of interaction and does not address the organizational, economic, and ethical dimensions of telemedicine that determine its effectiveness at the health-system level.

At the same time, a systematic review by Gonçalves et al [26] demonstrated that evidence regarding the usability of telemedicine systems from the perspective of health care professionals remains limited, and standardized, validated instruments for such evaluation are lacking.

These limitations point to the need to expand telemedicine evaluation beyond the purely user-experience domain, incorporating clinical, organizational, economic, and legal dimensions. A multidimensional and robust assessment requires a methodology that captures both the micro-level perspective of users and the macro-level processes of telemedicine implementation within the health care system. However, due to the constraints described above, the TUQ cannot be used as a standalone tool for comprehensive telemedicine assessment, making it necessary to complement it with a framework that encompasses systemic and organizational aspects. Model for Assessment of Telemedicine (MAST) is one of the most widely recognized international models for the comprehensive evaluation of telemedicine programs and is used across health care systems in countries with varying degrees of digital maturity. Given the limitations of existing tools, which predominantly focus on user-related aspects and do not address the clinical and system-level components of telehealth implementation, an approach capable of analyzing telemedicine at a broader level is required.

To address this gap, the study integrated the MAST as proposed by Kidholm et al [27]. MAST provides a structured approach to evaluating telemedicine solutions across 7 domains—from clinical effectiveness and safety to organizational, socioethical, and legal aspects. The combination of the TUQ and MAST models enabled the development of an instrument that merges the psychometric precision of user-experience assessment with a system-level framework for analyzing the organizational maturity of telemedicine.

The TUQ provides an in-depth understanding of the perception and usability of telemedicine systems, whereas the MAST framework encompasses the clinical, organizational, economic, and socioethical aspects of their implementation. These models complement each other: the TUQ reflects the micro-level (user experience), while MAST captures the macro-level (effectiveness and implementation conditions). This methodological combination delivers a holistic, multi-level approach that cannot be achieved using either instrument independently.

This combination makes it possible to conduct a comprehensive evaluation of telemedicine that accounts for both the subjective experiences of physicians and the systemic parameters of telemedicine service functioning. Such an approach is particularly important for countries undergoing active digital health development, including Kazakhstan.

In Kazakhstan, digital health is developing at the intersection of international standards and national specificities, which necessitates the use of adapted evaluation tools that reflect the real clinical and organizational context. This makes it essential to use instruments capable of capturing both physicians’ subjective perceptions and the operational characteristics of telemedicine services. Despite the expanding national telemedicine network and the active introduction of digital solutions, the country lacked validated instruments capable of systematically assessing physicians’ perceptions and the effectiveness of telemedicine. This created a methodological gap and limited the ability to monitor the quality of telemedicine programs.

The TUQ-MAST-KZ developed in this study represents the first instrument adapted to the context of Kazakhstan and the first to integrate the psychometric structure of TUQ with the systemic MAST framework, making it a unique example of such model integration within Central Asia.

Although the need for comprehensive telemedicine assessment tools is not unique to Kazakhstan and is shared by many countries with comparable levels of digital health maturity, the present instrument was developed and validated within the health care system of Kazakhstan.

Kazakhstan is characterized by a centralized health care system, an actively developing national telemedicine network, a multilingual clinical environment (Kazakh and Russian), and ongoing digital health reforms aligned with international standards. These characteristics informed the development, linguistic adaptation, and initial psychometric validation of the instrument within this national context, thereby defining its designation as the Kazakhstan version.

At the same time, TUQ-MAST-KZ is not intended for exclusive use within a single country. Its conceptual structure allows for further cross-country adaptation and validation in health care systems with similar organizational characteristics and levels of digital health development.

Consequently, TUQ-MAST-KZ bridges a critical gap between user perception and the evaluation of telemedicine implementation effectiveness, providing a comprehensive approach essential for advancing digital health in Kazakhstan.

The aim of this study was to develop and validate TUQ-MAST-KZ, the Kazakhstan version of the questionnaire integrating elements of the TUQ and MAST models for a comprehensive assessment of physicians’ perceptions and the effectiveness of telemedicine services.

## Methods

### Ethical Considerations

The study was conducted in accordance with the principles of the Declaration of Helsinki (1964 and subsequent revisions) and the national regulations of the Republic of Kazakhstan. The study protocol was reviewed and approved by the Local Bioethics Commission of the South Kazakhstan Medical Academy, Shymkent, Kazakhstan (protocol 3; approval date: April 25, 2025). All participants were informed about the purpose of the study and provided electronic informed consent prior to completing the questionnaire. No confidential or identifiable data were entered into any automated systems. The participants did not receive any financial or nonfinancial compensation.

### Study Design

This study used a multiphase methodological and descriptive design (multiphase, nonexperimental, cross-sectional study) aimed at developing, culturally adapting, and psychometrically validating a new instrument—the TUQ-MAST-KZ questionnaire—intended to assess physicians’ perceptions, usability, and perceived effectiveness of telemedicine services in Kazakhstan.

The study consisted of several sequential stages: (1) literature analysis and selection of the methodological framework (TUQ and MAST), (2) development and structuring of the questionnaire, (3) linguistic and cultural adaptation, (4) expert evaluation of content validity (CVI), and (5) pilot testing and analysis of internal reliability (Cronbach α).

Such a multiphase approach ensured the scientific rigor, transparency, and reproducibility of the instrument development process.

### Instrument Development: Theoretical Framework (TUQ and MAST)

The methodological foundation of the TUQ-MAST-KZ questionnaire was based on the integration of two internationally recognized models for telemedicine evaluation:

1. TUQ [25]: an instrument designed to assess users’ perceptions, usability, interface quality, and satisfaction with telemedicine technologies.
2. MAST [27]: a comprehensive assessment framework that includes seven domains: clinical, organizational, economic, ethical, legal, social, and technological.

The integration of these models made it possible to create a multilevel instrument that combines user-focused and system-level aspects of telemedicine evaluation. Based on TUQ and MAST, the TUQ-MAST-KZ questionnaire was developed, with its structure summarized in the ”Questionnaire Construction” section.

### Questionnaire Construction

The development of the TUQ-MAST-KZ questionnaire was based on an analysis of international telemedicine evaluation instruments, including the TUQ and MAST models. The initial set of items was formulated using the conceptual components of these models.

The draft version of the questionnaire was reviewed by an interdisciplinary expert panel to evaluate the logical structure, eliminate duplication, verify the accuracy of item wording, and ensure alignment with the domains of the MAST model. The experts’ comments focused on refining terminology, removing overlapping items, and adapting statements to the clinical and organizational context of Kazakhstan.

The final version of the TUQ-MAST-KZ questionnaire included 27 items, including:

- 13 Likert scale statements (5-point scale ranging from 1=“strongly disagree” to 5=“strongly agree”)
- 4 dichotomous questions (“yes/no” responses)
- 8 categorical questions reflecting demographic and professional characteristics of respondents
- 2 open-ended questions for additional comments and descriptions of individual experience

This structure made it possible to balance quantitative and qualitative data and to ensure a comprehensive assessment of physicians’ perceptions, experiences, and conditions of telemedicine use in Kazakhstan.

The full version of the TUQ-MAST-KZ questionnaire, including all 27 items and their distribution across the adapted TUQ and MAST domains, is provided in Multimedia Appendix 1.

### Linguistic and Cultural Adaptation

The linguistic adaptation was applied to the final version of the TUQ-MAST-KZ instrument. The adaptation process followed the international ISPOR (The Professional Society for Health Economics and Outcomes Research) guidelines for translation and cultural adaptation of assessment instruments [28]. The process consisted of four key stages:

1. Forward translation of the final set of TUQ-MAST-KZ items into Kazakh and Russian, performed by two independent professional translators with experience in medicine and digital health.
2. Reconciliation of translations during a meeting of an interdisciplinary expert panel, which included a physician, a public health specialist, a faculty member, and an IT specialist.
3. Back translation of both language versions into English by an independent linguist unfamiliar with the original instrument.
4. Comparative analysis of the original author-developed TUQ-MAST-KZ version and the back translations to ensure conceptual, semantic, and content equivalence.

The final Kazakh and Russian versions of the TUQ-MAST-KZ were deemed by experts to be fully consistent with the original author-developed version of the instrument, ensuring its correct application among physicians proficient in different languages.

### Expert Validation

The content validity of the questionnaire was assessed using an expert evaluation approach with the participation of 6 independent experts. Each item was evaluated according to three criteria—relevance, clarity of wording, and necessity—using a 4-point scale, where 1 indicated “not relevant” and 4 indicated “highly relevant.”

The calculation of the individual content validity index (I-CVI) and the scale-level content validity index (S-CVI/Ave) was performed in accordance with the recommendations of Polit and Beck [29] and Lynn [30]. A number of experts met the international minimum requirements for content validation.

According to the results, all items had an I-CVI of ≥0.80, and the overall index was S-CVI/Ave=0.94, indicating high agreement among experts and excellent content validity of the questionnaire.

All calculations were performed manually and additionally verified in IBM SPSS (Statistical Package for the Social Sciences) Statistics (version 27.0) using the *Descriptive Statistics* function.

The experts confirmed that the content and phrasing of the items accurately reflect the intended construct areas and correspond to the structure of the MAST model.

### Pilot Testing and Data Collection

The pilot online study was conducted in the Republic of Kazakhstan from May 30, 2025, to June 9, 2025, using a secure electronic survey form (Google Forms). A total of 156 physicians from various regions and levels of health care participated in the survey. The sample included general practitioners, internists, cardiologists, oncologists, surgeons, and other specialists representing both urban and rural health care facilities.

Participation was voluntary and anonymous. All respondents were informed about the purpose of the study and provided electronic informed consent.

### Inclusion Criteria

The study included physicians working in public or private health care organizations in Kazakhstan, representing different levels of care (primary, secondary, and tertiary) and both urban and rural regions. Participants were required to be fluent in Kazakh or Russian and to agree to participate in the study.

The purpose of the pilot phase was to assess the applicability and clarity of the TUQ-MAST-KZ questionnaire, as well as to evaluate its usability in clinical practice. Based on the pilot results, several item wordings were refined to improve clarity and ensure better adaptation of the instrument to the health care context of Kazakhstan.

The pilot study was not intended to analyze factors influencing telemedicine perception. Its sole purpose was to test the functionality of the structure, content consistency, and internal reliability of the questionnaire. The final version of the TUQ-MAST-KZ includes 27 statements approved for the main phase of the study.

All collected data are stored in anonymized form and can be provided to the editorial office upon request.

### Statistical Analysis

Data were analyzed using IBM SPSS Statistics (version 27.0) and Microsoft Excel (version 16.0). The analysis was performed using standard SPSS procedures—*Reliability Analysis* and *Descriptive Statistics*, which ensures the reproducibility of all computations.

To assess the internal consistency of the TUQ-MAST-KZ questionnaire, the Cronbach α was calculated. The interpretation of Cronbach α values followed the recommendations of Nunnally and Bernstein [31], where Cronbach *α*≥0.90 indicates excellent internal consistency. For the final version of the instrument, the Cronbach *α* coefficient was 0.93.

Frequencies, percentages, means, and SDs were used to describe the sample. During the pilot phase, SPSS was additionally used to evaluate the response distributions and basic domain-level indicators, allowing verification of item performance before the main stage of the study.

All statistical procedures followed international standards for reporting psychometric validation of questionnaires [29,32]. The significance level was set at *P*<.05.

A detailed quantitative analysis of physicians’ perceptions of telemedicine and comparisons across respondent groups will be presented in a separate publication focusing on the main phase of TUQ-MAST-KZ implementation.

## Results

This section presents the quantitative findings from the pilot validation of the TUQ-MAST-KZ questionnaire, including sample characteristics, content validity indicators, and the instrument’s internal consistency metrics.

### Sample Characteristics

A total of 156 physicians were included in the analysis, representing different types of health care organizations and levels of urbanization. Among the participants, 62.2% (n=97) were women and 37.8% (n=59) were men. The age distribution was as follows: 23 to 30 years (n=50, 32.1%), 31 to 40 years (n=40, 25.6%), 41 to 50 years (n=24, 15.4%), 51 to 60 years (n=36, 23.1%), and over 60 years (n=6, 3.8%).

Professional experience was distributed as follows: 3 to 5 years (n=63, 40.4%), more than 20 years (n=52, 33.3%), 6 to 10 years (n=16, 10.3%), 11 to 15 years (n=12, 7.7%), and 16 to 20 years (n=13, 8.3%).

By type of medical organization, respondents represented polyclinics (n=70, 44.9%), hospitals (n=43, 27.6%), medical centers (n=25, 16%), medical universities (n=8, 5.1%), health administration bodies (n=4, 2.6%), dispensaries (n=1, 0.6%), and other institutions (n=5, 3.2%).

By level of urbanization of the workplace, participants were distributed as follows: metropolitan areas (n=87, 55.8%), large cities and urbanized zones (n=29, 18.6%), small towns (n=15, 9.6%), and rural areas (n=25, 16%).

### Professional Profiles

Participants represented a wide range of clinical specialties. The largest groups included general practitioners (n=37, 23.7%), surgeons (n=17, 10.9%), otorhinolaryngologists (n=12, 7.7%), internists (n=11, 7.1%), and oncologists (n=10, 6.4%). Other specialties were represented in smaller proportions.

The linguistic and cultural adaptation of the questionnaire did not require substantial modifications.

I-CVIs ranged from 0.83 to 1.00, and the overall S-CVI/Ave was 0.94.

### Pilot Testing

The pilot testing of the final version of TUQ-MAST-KZ was conducted among 156 physicians from various regions of Kazakhstan. The average completion time was approximately 10 minutes, indicating that the questionnaire is convenient to use and suitable for real-world clinical workloads.

During the analysis of the pilot data, several items were identified as requiring minor wording adjustments to improve clarity; these revisions were editorial in nature and did not affect the structure of the instrument. All items maintained logical coherence, and the sequence of domains was perceived by respondents as intuitive and easy to follow.

The questionnaire did not require substantial modifications—its structure, overall length, and item phrasing were found to be appropriate for clinical practice. The final version of TUQ-MAST-KZ includes 27 items and was used in the main stage of the study. The anonymized pilot data confirmed the technical correctness of the format and the absence of difficulties during completion.

### Reliability (Cronbach α)

The internal consistency of the TUQ-MAST-KZ scale was evaluated using Cronbach α. The analysis included 10 Likert scale items (Q10, Q12-Q15, Q17-Q18, and Q19-Q21) corresponding to the key domains of the instrument. The overall Cronbach α coefficient was 0.924, indicating a high level of internal consistency.

The domain-level analysis confirmed the stability of the instrument’s structural components:

1. Effectiveness and clinical applicability (Q12-Q15): *α*=0.879
2. Reliability and technical aspects (Q17-Q18): *α*=0.811
3. Quality of interaction (Q19-Q21): *α*=0.840

All values exceed the commonly accepted threshold of *α*≥0.70, indicating high reliability of the overall scale and its individual domains. The calculations were performed in IBM SPSS Statistics (version 27.0).

Only the Likert-type items, which form the multicomponent structure of the questionnaire, were used to assess internal consistency. Nonscale items (demographic, categorical, and open-ended questions) were excluded from the analysis, as they do not reflect latent constructs related to the perception of telemedicine (Table 1).

**Table 1.** Internal consistency (Cronbach α) of the TUQ-MAST-KZ^a^ scale.

Domain	Items	Cronbach α	Interpretation
Effectiveness and clinical applicability	Q12-Q15	0.879	High consistency
Reliability and technical aspects	Q17-Q18	0.811	Good consistency
Quality of interaction	Q19-Q21	0.840	Good consistency
Overall scale	Q10, Q12-Q15, and Q17-Q21	0.924	Excellent consistency

a TUQ-MAST-KZ: Kazakhstan version of the questionnaire based on the Telehealth Usability Questionnaire and Model for Assessment of Telemedicine models

The final version of TUQ-MAST-KZ includes 27 items distributed across thematic areas that reflect key components of the MAST model and the clinical context of telemedicine use. The structure of the questionnaire was refined based on expert review and pilot testing, ensuring logical coherence, the absence of redundancy, and alignment with the national characteristics of Kazakhstan’s health care system.

A separate section of the questionnaire includes 9 items designed to collect demographic and professional characteristics of respondents (Q1-Q9). This block is not part of the MAST model domains; however, it is essential for describing the sample and analyzing between-group differences.

The questionnaire contains 4 types of items: 13 Likert scale statements (1-5), 4 dichotomous items, 8 categorical questions, and 2 open-ended questions. The Likert scale statements form a multicomponent construct of physicians’ perceptions of telemedicine services and serve as the basis for psychometric evaluation.

TUQ-MAST-KZ was designed to assess those domains of the MAST model that can be reliably and appropriately measured through physician-reported survey methods. The instrument covers technological characteristics, clinical applicability, reliability and safety, organizational aspects, and the subjective perception of telemedicine services—components that directly reflect the practical experience of health care professionals (Table 2).

**Table 2.** Final structure of the TUQ-MAST-KZ^a^ questionnaire by domains.

Domain category	Basis	Construct content	TUQ-MAST-KZ items
Technological characteristics	MAST^b^: technological aspects	Ease of use, efficiency, and perceived impact on quality of care	Q12-Q15
Clinical applicability	MAST: clinical aspects	Clinical relevance and applicability of telemedicine The feasibility of using telemonitoring in daily practice	Q16
Safety and technical reliability	MAST: safety or technical reliability	Technical stability, errors, and perceived risks	Q17-Q18
Subjective perception and interaction	TUQ^c^: usability or interaction quality	Comfort, ease of interaction, and quality of communication	Q10 and Q19-Q21
Organizational context	MAST: organizational aspects	Organizational support, involvement, and availability of necessary conditions	Q11 and Q24-Q25
Organizational readiness and acceptance	MAST: sociotechnical or organizational readiness	Perceived potential for further development of telemedicine and willingness to support its implementation	Q22-Q23
Qualitative feedback (author-defined domain)	Author modification	Comments, barriers, and subjective suggestions	Q26-Q27

a TUQ-MAST-KZ: Kazakhstan-adapted questionnaire integrating components of the Telehealth Usability Questionnaire and Model for Assessment of Telemedicine models.

b MAST: Model for Assessment of Telemedicine.

c TUQ: Telehealth Usability Questionnaire.

The economic, legal, and socioethical elements of the MAST model were intentionally not included in the structure of the questionnaire, as their assessment requires specialized expert-based methodologies and lies beyond the scope of self-report survey instruments.

TUQ-MAST-KZ demonstrated a stable structure and good acceptability among physicians. The pilot testing confirmed the logical organization of domains, the absence of item redundancy, and the relevance of the questions to the clinical context of telemedicine practice in Kazakhstan. The instrument covers technological, clinical, and organizational aspects and provides a comprehensive description of physicians’ experience with telemedicine services.

## Discussion

### Principal Findings

A questionnaire, TUQ-MAST-KZ, was developed and validated to provide a comprehensive assessment of telemedicine from the perspective of physicians. The integration of the TUQ and MAST models made it possible to combine measures of usability and perception with key organizational and clinical aspects of telemedicine implementation.

During development, a rigorous content-validation methodology was followed: the questionnaire items were mapped to the relevant domains of the MAST model, ensuring a coherent structure and construct alignment. The Cronbach α coefficient of 0.93 indicates high internal reliability, comparable to international adaptations of the TUQ conducted in Germany [33], Thailand [34], and Denmark [35].

The linguistic and cultural adaptation was carried out in accordance with the recommendations of Wild et al [28], following established principles of content validation [29,30]. The forward and backward translations, expert reconciliation, and refinement of item wording ensured the instrument’s appropriateness and accuracy for use in the Kazakhstani context.

The relevance of using the TUQ is supported by findings from recent systematic reviews. Özkeskin et al [36] demonstrated the importance of applying validated instruments to assess user experience in telemedicine. Nouri et al [37] highlighted the critical role of digital literacy; the high proportion of respondents in our study (70.5%) who reported a need for training reinforces this conclusion.

The development of TUQ-MAST-KZ addresses a significant gap in the tools available for assessing physicians’ perceptions of telemedicine in Kazakhstan. The instrument can be used in studies of digital maturity within health care organizations, professional development programs, internal audits of telemedicine services, and during the implementation of new digital solutions. It also has the potential to be adapted for use in other Central Asian countries and further expanded—including the development of a patient-reported version.

Unlike previously developed instruments such as TSQ, Patient Assessment of Communication during Telemedicine Questionnaire, and Service User Technology Acceptability Questionnaire [18,20,21], which are primarily focused on assessing patient experience, the TUQ-MAST-KZ combines usability and perception metrics with components that reflect the organizational and clinical context of telemedicine practice. This makes the instrument more suitable for the systemic evaluation of telemedicine implementation. In the future, the development of a shortened version of the scale based on principal component analysis, as well as factor analysis on a larger sample, is planned. Thus, the TUQ-MAST-KZ can be recommended as a reliable, structurally valid, and practically applicable tool for assessing physicians’ perceptions of telemedicine services, adapted to the national context and aligned with international methodological standards.

### Conclusions

This study developed, culturally adapted, and conducted the initial validation of the TUQ-MAST-KZ questionnaire designed to assess physicians’ perceptions, usability, and perceived effectiveness of telemedicine services within the health care context of Kazakhstan. The instrument is based on the integration of the TUQ [25] and MAST [27] models, which allowed for the combination of user-centered and organizational dimensions of telemedicine assessment and ensured its relevance to the national context. The TUQ-MAST-KZ represents the first validated tool developed specifically to evaluate physicians’ perceptions of telemedicine services in Kazakhstan and is among the few instruments of this type available in Central Asia.

The pilot survey conducted among 156 physicians from various specialties confirmed the instrument’s high internal reliability (Cronbach *α*=0.93) and strong content validity. The data demonstrated generally positive perceptions of telemedicine platforms across several dimensions, highlighted the need for additional training among health care professionals, and revealed specific organizational factors requiring further improvement. These findings underscore the importance of implementing structured assessment tools in clinical practice to support the sustainable development of telemedicine.

The TUQ-MAST-KZ can be applied in studies assessing the digital maturity of health care organizations, in the planning of professional education programs, in monitoring the implementation of telemedicine services, and in future research examining health care professionals’ perceptions of digital technologies. The results obtained form a foundation for the broader application of the instrument and support the further development of the telemedicine digital maturity index (KazTeleHealthIndex), which will be presented in subsequent studies.

## Supplementary material

10.2196/80693 Multimedia Appendix 1 TUQ-MAST-KZ, a Kazakhstan version of the questionnaire based on the Telehealth Usability Questionnaire (TUQ) and Model for Assessment of Telemedicine (MAST) models, for assessing physicians’ perceptions and effectiveness of telemedicine services.

## Abbreviations

I-CVI: individual content validity index
ISPOR: The Professional Society for Health Economics and Outcomes Research
MAST: Model for Assessment of Telemedicine
S-CVI/Ave: scale-level content validity index
SPSS: Statistical Package for the Social Sciences
TSQ: Telemedicine Satisfaction Questionnaire
TUQ: Telehealth Usability Questionnaire
TUQ-MAST-KZ: Kazakhstan version of the questionnaire based on the Telehealth Usability Questionnaire and Model for Assessment of Telemedicine models
WHO: World Health Organization
