# Supplementary material for: Development and Validation of the Kazakhstan Version of the Questionnaire Based on the Telehealth Usability Questionnaire and Model for Assessment of Telemedicine Models for Evaluating the Usability and Effectiveness of Telemedicine Services Among Physicians: Multiphase Cross-Sectional Study
Source: JMIR Form Res. 2026 Feb 2;10:e80693. doi: 10.2196/80693 (PMC12863653; doi:10.2196/80693)
Supplement: Multimedia Appendix 1 [file formative-v10-e80693-s001.pdf]

Multimedia Appendix 1

Telehealth Usability Questionnaire – Model for Assessment of Telemedicine, Kazakhstan Version (TUQ-MAST-KZ)

This questionnaire was developed by integrating two internationally validated instruments:

TUQ – Telehealth Usability Questionnaire

MAST – Model for Assessment of Telemedicine

The TUQ-MAST-KZ questionnaire assesses usability, perceived effectiveness, and systemic factors of telemedicine services in the context of Kazakhstan's healthcare system.

A validated tool for assessing telemedicine usability and effectiveness among physicians.

Dear Colleagues,

The purpose of this survey is to gather healthcare professionals' perspectives on the current state, quality, and accessibility of telemedicine services, including video consultations and mobile health technologies. The collected data will inform evidence-based recommendations for improving the telemedicine system in Kazakhstan.

The objectivity of the analysis and the accuracy of the resulting recommendations depend on the completeness and sincerity of your responses. We kindly ask you to answer all questions independently and consistently, without omitting any. This questionnaire is anonymous, and all collected data will be used exclusively in aggregated form.

Thank you for your participation and contribution to the development of digital healthcare!

SECTION 1. GENERAL INFORMATION

What is your gender?

☐ Male

☐ Female

Your age

23–30

31–40

41–50

51–60

over 60

What type of medical institution do you work in?

Polyclinic

Hospital

Public Health Department

Dispensary

Medical center

Other: ___________

Your work experience:

3-5 years

6-10 years

11-15 years

16-20 years

over 20 years

5. What is the location of your place of practice:

Village / Rural area

Town / Small city

Large city / Urban area

Metropolis

What is your medical specialty? (please specify): ________________

7. How familiar are you with telemedicine technologies?

1 (Not familiar at all)

2 (Slightly familiar)

3 (Moderately familiar)

4 (Well familiar)

5 (Very familiar)

Which telemedicine format do you use most often?

☐ National Telemedicine Network

☐ video consultations

☐ phone

☐ mobile applications

☐ chats/messengers

☐ wearable devices (telemonitoring)

☐ other: ____________________

Have you used telemedicine technologies that involve artificial intelligence (AI)

(e.g., automated ECG analysis, AI-based image diagnostics, chatbots, exacerbation prediction, etc.)?

☐ Yes, regularly

☐ Sometimes

☐ No

☐ I don’t know / Not sure

SECTION 2. CONVENIENCE AND LEARNABILITY

Telemedicine platforms are easy and intuitive to use.

☐ 1 – Strongly disagree

☐ 2 – Disagree

☐ 3 – Neutral

☐ 4 – Agree

☐ 5 – Strongly agree

Do you feel that you need additional training to use telemedicine platforms effectively?

☐ Yes

☐ No

☐ Not sure / I find it difficult to answer

SECTION 3. EFFICACY AND CLINICAL APPLICABILITY

Telemedicine helps me monitor the patient’s condition in a timely and effective manner.

☐ 1 – Strongly disagree

☐ 2 – Disagree

☐ 3 – Neutral

☐ 4 – Agree

☐ 5 – Strongly agree

13. Telemonitoring (if used) allows for earlier detection of patient deterioration.

☐ 1 – Not effective at all

☐ 2 – Slightly effective

☐ 3 – Moderately effective

☐ 4 – Quite effective

☐ 5 – Highly effective

14. Telemedicine is effective in the treatment of patients with chronic diseases.

☐ 1 – Not effective at all

☐ 2 – Slightly effective

☐ 3 – Moderately effective

☐ 4 – Quite effective

☐ 5 – Highly effective

15. Remote formats have reduced the need for patients to visit healthcare facilities in person.

☐ 1 – Not effective at all

☐ 2 – Slightly effective

☐ 3 – Moderately effective

☐ 4 – Quite effective

☐ 5 – Highly effective

16. Would you recommend the use of telemedicine for certain categories of patients (e.g., chronic cases, remote areas)?

☐ Yes

☐ No

☐ Not sure / I find it difficult to answer

SECTION 4. RELIABILITY AND TECHNICAL DIFFICULTIES

17. The connection when using telemedicine platforms is stable and reliable.

☐ 1 – Strongly disagree

☐ 2 – Disagree

☐ 3 – Neutral

☐ 4 – Agree

☐ 5 – Strongly agree

18. Data transfer from wearable devices (telemonitoring) functions correctly and without errors.

☐ 1 – Strongly disagree

☐ 2 – Disagree

☐ 3 – Neutral

☐ 4 – Agree

☐ 5 – Strongly agree

Section 5. Quality of interaction

19. I am able to establish effective contact with patients through telemedicine channels.

☐ 1 – Strongly disagree

☐ 2 – Disagree

☐ 3 – Neutral

☐ 4 – Agree

☐ 5 – Strongly agree

20. Patients feel comfortable communicating through remote consultation formats.

☐ 1 – Strongly disagree

☐ 2 – Disagree

☐ 3 – Neutral

☐ 4 – Agree

☐ 5 – Strongly agree

21. The remote format does not negatively affect the quality of my communication with patients.

☐ 1 – Strongly disagree

☐ 2 – Disagree

☐ 3 – Neutral

☐ 4 – Agree

☐ 5 – Strongly agree

SECTION 6. FUTURE USE AND READINESS

22. How do you assess the future potential for the development of telemedicine in your region or country?

☐ 1 – Very low

☐ 2 – Low

☐ 3 – Moderate

☐ 4 – High

☐ 5 – Very high

23. How ready are you to recommend the implementation of telemonitoring at the national level?

☐ 1 – Very low

☐ 2 – Low

☐ 3 – Moderate

☐ 4 – High

☐ 5 – Very high

SECTION 7. ORGANIZATION AND MANAGEMENT SUPPORT OF TELEMEDICINE:

24. Does your medical facility have established guidelines or protocols for providing telemedicine services?

☐ Yes

☐ No

☐ Not sure / I find it difficult to answer

25. Who is responsible for organizing and ensuring the quality of telemedicine consultations in your institution?

☐ Attending physician

☐ Department management

☐ Specially appointed coordinator

☐ No one is specifically assigned

SECTION 8. PROPOSALS AND BARRIERS

26. Which telemedicine topics would you like to explore further to enhance your professional qualifications?

____

27. In your opinion, what should be improved to support the development of telemedicine and telemonitoring in Kazakhstan?

____
